# Supplementary material for: Elucidating the mitochondrial proteome of Toxoplasma gondii reveals the presence of a divergent cytochrome c oxidase
Source: eLife. 2018 Sep 11;7:e38131. doi: 10.7554/eLife.38131 (PMC6156079; doi:10.7554/eLife.38131)
Supplement: Source code 1. [file elife-38131-code1.pdf]

```
setwd("/Users/Esther/Documents/Toxo/Seahorse/R analysis")
seahorse=read.csv("mOCR IMP1_seahorse.csv")
seahorse$Plate=factor(seahorse$Plate)
seahorse$Group=factor(seahorse$Group)
seahorse$Well=factor(seahorse$Well)

#visualize raw data
library(ggplot2)
ggplot(seahorse,aes(Group,OCR,colour=Group))+geom_point()
+xlab("Group")+ylab("OCR")
ggplot(seahorse,aes(Time,OCR,colour=Group))+geom_point()
+xlab("Time")+ylab("OCR")
seahorse=seahorse[seahorse$OCR>0,]
ggplot(seahorse,aes(Group, OCR, colour=Group))+geom_boxplot()
ggplot(seahorse,aes(Time,OCR,colour=Group))+geom_boxplot()
+xlab("Time")+ylab("OCR")

#Fit a linear mixed-effects model to the dataset
library(lmerTest)
library(pbkrtest)
library(emmeans)
model1=lmer(OCR~Group+(1|Plate)+(1|Plate:Well),data=seahorse)
anova(lm(OCR~Group+Time,data=seahorse))
anova(model1)
summary(model1)
results=lsmeans(model1,pairwise~Group)

#plot data
library(broom)
summarisedinfo=tidy(lsmeans::lsmeans(model1,"Group"))
write.csv(summarisedinfo,"seahorse1.csv")
library(ggsignif)
ggplot(summarisedinfo,aes(Group,estimate,fill=Group)) +
geom_bar(stat="identity") + geom_errorbar(aes(ymin=estimate-
std.error,ymax=estimate+std.error),width=.3) + xlab("Group")
+ylab("OCR")

#pairwise comparison for statistical analysis
results
```
