# Supplementary material for: Elucidating the mitochondrial proteome of Toxoplasma gondii reveals the presence of a divergent cytochrome c oxidase
Source: eLife. 2018 Sep 11;7:e38131. doi: 10.7554/eLife.38131 (PMC6156079; doi:10.7554/eLife.38131)
Supplement: Source code 2. [file elife-38131-code2.pdf]

```
#(no rows with 0)
source("https://bioconductor.org/biocLite.R")
biocLite("edgeR")
library(edgeR)
library(limma)

setwd("C:/Users/Linden.Vieng-on-HP/Desktop/Experiments/R/MS
analysis")
ms_data<-read.csv("CoIP results.csv")
str(ms_data)

counts<-ms_data[,6:11]
rownames(counts)<-ms_data$Accession.Number
group<-factor(c(2,2,2,1,1,1))

y<-DGEList(counts=counts, group=group)
#Calculate the normalisation factors. Consider leaving out this
step?
y<-calcNormFactors(y)
design<-model.matrix(~group)
y<-estimateDisp(y,design)
fit<-glmFit(y,design)
#Query the fit object, note: 2nd column are estimates of log(fold
change)
fit$coefficients

#Likelihood Ratio tests. coef=2 means that we are interested in
group differences
lrt<-glmLRT(fit,coef=2)
topTags(lrt,30)

lrt_table<-lrt$table
logFC10<-subset(lrt_table,logFC>=10)
logFC10[order(logFC10$PValue),]

#Make Volcano Plot
#http://www.gettinggeneticsdone.com/2014/05/r-volcano-plots-to-
visualize-rnaseq-microarray.html
with(lrt_table, plot(logFC, -log10(PValue), pch=20,
                     main="Final normalised Volcano plot with 0
values excluded", xlim=c(-15,15)))
# Add colored points: red if padj<0.05, orange of log2FC>1, green if
both)
with(subset(lrt_table,PValue<.05 ), points(logFC, -log10(PValue),
pch=20, col="red"))
with(subset(lrt_table, abs(logFC)>log(5)), points(logFC, -
log10(PValue), pch=20, col="orange"))
with(subset(lrt_table, PValue<.05 & abs(logFC)>log(5)),
     points(logFC, -log10(PValue), pch=20, col="green"))

write.csv(lrt_table,"volcanoplotexport.csv")
```
