# Supplementary material for: Elucidating the mitochondrial proteome of Toxoplasma gondii reveals the presence of a divergent cytochrome c oxidase
Source: eLife. 2018 Sep 11;7:e38131. doi: 10.7554/eLife.38131 (PMC6156079; doi:10.7554/eLife.38131)
Supplement: Transparent reporting form [file elife-38131-transrepform.pdf]

eLIFE

1st Floor
24 Hills Road
Cambridge CB2 1JP, UK

P 01223 855340
W elifesciences.org
T @elife

eLife's transparent reporting form

We encourage authors to provide detailed information *within their submission* to facilitate the interpretation and replication of experiments. Authors can upload supporting documentation to indicate the use of appropriate reporting guidelines for health-related research (see EQUATOR Network), life science research (see the BioSharing Information Resource), or the ARRIVE guidelines for reporting work involving animal research. Where applicable, authors should refer to any relevant reporting standards documents in this form.

If you have any questions, please consult our Journal Policies and/or contact us: editorial@elifesciences.org.

Sample-size estimation

- You should state whether an appropriate sample size was computed when the study was being designed
- You should state the statistical method of sample size computation and any required assumptions
- If no explicit power analysis was used, you should describe how you decided what sample (replicate) size (number) to use

Please outline where this information can be found within the submission (e.g., sections or figure legends), or explain why this information doesn't apply to your submission:

We did not undertake any power analyses prior to the experimental design. Unless otherwise stated, we undertook a minimum of three biological replicates for each quantitative experiment, following conventions in the field.

Replicates

- You should report how often each experiment was performed
- You should include a definition of biological versus technical replication
- The data obtained should be provided and sufficient information should be provided to indicate the number of independent biological and/or technical replicates
- If you encountered any outliers, you should describe how these were handled
- Criteria for exclusion/inclusion of data should be clearly stated
- High-throughput sequence data should be uploaded before submission, with a private link for reviewers provided (these are available from both GEO and ArrayExpress)

Please outline where this information can be found within the submission (e.g., sections or figure legends), or explain why this information doesn't apply to your submission:

eLife Sciences Publications, Ltd is a limited liability non-profit non-stock corporation incorporated in the State of Delaware, USA, with company number 5030732, and is registered in the UK with company number FC030576 and branch number BR015634 at the address 1st Floor, 24 Hills Road, Cambridge CB2 1JP | August 2014

How often was each experiment performed? Specifying the number of replicates

Mitochondrial proteomics

Lines 810-813. Description of the number of repeats of the APEX and BirA* proteomics.

*Analysis of the r*Tg*ApiCox25 mutant*

Figure 5B legend. Description of replicates of plaque assays.
Figure 5C legend. Description number of plaques measured for box and whisker plot.
Figure 5D legend. Description of number of independent experiments performed for measuring mitochondrial oxygen consumption rate (mOCR).
Lines 900-902. Description of number of wells used for background correction and number of technical replicates undertaken for each biological replicate in the XFe96 analyzer experiments.
Figure 5E legend. Description of number of independent experiments performed for measuring mOCR vs extracellular acidification rate.
Figure 5 – figure supplement 2 legend. Description of the number of independent experiments undertaken to calculate maximal and spare capacity mOCR.
Figure 5 – figure supplement 3A legend. Description of the number of vacuoles analyzed for microscopy analysis of mitochondrial morphology, and the number of biological replicates performed for this experiment.
Figure 5 – figure supplement 3B legend. Description of the number of independent plaque assays performed to analyze cell viability in r*Tg*ApiCox25 parasites.

Analysis of the T. gondii *COX complex*

Figure 6C legend. Description of the number of repeats for the *Tg*ApiCox25 and *Tg*Tom40 immunoprecipitations.

Definitions of biological vs technical replication

All biological replicates that we describe in the manuscript were performed on different days, using independent parasite sub-cultures. Technical replicates in the XFe96 flux analyzer experiments were performed in different wells of the same plate on the same day. In the terminology used in our manuscript, we refer to 'biological replicates' as independent experiments. The numbers of biological and/or technical replicates undertaken for each experiment are reported in the figure legends or relevant methods section, as outlined above.

Outliers

Not applicable.

Criteria for inclusion/exclusion of data

Line 994. For the oxygen consumption rate (OCR) assays, wells that yielded negative OCR values were excluded from the final analysis.
Lines 1003-1004. For the analysis of proteins in Figure 6C, only proteins identified in both experimental conditions and each biological replicate were included in the final analysis.

High throughput sequence data

Not applicable.

eLife Sciences Publications, Ltd is a limited liability non-profit non-stock corporation incorporated in the State of Delaware, USA, with company number 5030732, and is registered in the UK with company number FC030576 and branch number BR015634 at the address 1st Floor, 24 Hills Road, Cambridge CB2 1JP | August 2014

Statistical reporting

- Statistical analysis methods should be described and justified
- Raw data should be presented in figures whenever informative to do so (typically when N per group is less than 10)
- For each experiment, you should identify the statistical tests used, exact values of N, definitions of center, methods of multiple test correction, and dispersion and precision measures (e.g., mean, median, SD, SEM, confidence intervals; and, for the major substantive results, a measure of effect size (e.g., Pearson's r, Cohen's d)
- Report exact p-values wherever possible alongside the summary statistics and 95% confidence intervals. These should be reported for all key questions and not only when the p-value is less than 0.05.

Please outline where this information can be found within the submission (e.g., sections or figure legends), or explain why this information doesn't apply to your submission:

eLife Sciences Publications, Ltd is a limited liability non-profit non-stock corporation incorporated in the State of Delaware, USA, with company number 5030732, and is registered in the UK with company number FC030576 and branch number BR015634 at the address 1st Floor, 24 Hills Road, Cambridge CB2 1JP | August 2014

Description of statistical methods and tests

Mitochondrial proteomics

Figure 2 legend (also Lines 166-168). Description of statistical 'cut-offs' used to define the mitochondrial proteome.

Lines 854-859. Description of data analysis for the quantitative proteomics pipeline.

Mitochondrial proteome data analysis

Supplementary File 2 legend (see also Lines 190-191). Description of *p* value cutoff for metabolic pathway enrichment analysis.

Analysis of the rTgApiCox25 mutant

Figure 5C legend. Description of box and whisker plots to analysis plaque sizes.
Figure 5D legend. Description of error bars and statistical analysis for mOCR assays.
Lines 998-1001. Description of linear mixed-effects models used in the XFe96 experiments. The R code used for the analysis is included as Source Code File 1.
Figure 5E legend. Description of error bars in mOCR vs ECAR analysis.
Lines 996-1001. Description of statistical treatment of the mOCR and ECAR data.
Figure 5 – figure supplement 2 legend. Description of error bars and statistical analysis for maximal and spare capacity mOCR analysis.

Analysis of the T. gondii COX complex proteomics

Lines 1001-1006. Description of the data analysis pipeline used for analyzing proteins identified from the *Tg*ApiCox25 and *Tg*Tom40 immunoprecipitations.
Figure 6C legend. Description of the statistical analysis of proteins identified from the *Tg*ApiCox25 and *Tg*Tom40 immunoprecipitations.

Presenting raw data

Supplementary File 1 includes a list of all peptides identified in the mitochondrial proteomics. This file also lists the calculated $\log_2$ protein ratios and *p* values used in generating Figure 2A-B.

The categorization of proteins used in generating Figures 2D and 4 are included in Supplementary Files 1 and 3.

Supplementary File 5 lists all of the proteins, and the intensities of each, identified in the co-immunoprecipitation proteomics. This file also lists the calculated logFC and *p* values used in generating Figure 6C.

Listing *p* values

The *p* values that are plotted on the y axes of the volcano plots shown in Figures 2A-B and 6C are included in Supplementary Files 1 and 5.

The *p* values obtained when statistically comparing the various relevant experimental conditions in the XFe96 experiments are noted in Figure 5D and Figure 5 – figure supplement 2.

(For large datasets, or papers with a very large number of statistical tests, you may upload a single table file with tests, Ns, etc., with reference to sections in the manuscript.)

Group allocation

- Indicate how samples were allocated into experimental groups (in the case of clinical studies, please specify allocation to treatment method); if randomization was used, please also state if restricted randomization was applied
- Indicate if masking was used during group allocation, data collection and/or data analysis

Please outline where this information can be found within the submission (e.g., sections or figure legends), or explain why this information doesn't apply to your submission:

Not applicable

Additional data files ("source data")

- We encourage you to upload relevant additional data files, such as numerical data that are represented as a graph in a figure, or as a summary table
- Where provided, these should be in the most useful format, and they can be uploaded as "Source data" files linked to a main figure or table
- Include model definition files including the full list of parameters used
- Include code used for data analysis (e.g., R, MatLab)
- Avoid stating that data files are "available upon request"

Please indicate the figures or tables for which source data files have been provided:

eLIFE
1st Floor
24 Hills Road
Cambridge CB2 1JP, UK
P 01223 855340
W elifesciences.org
T @elife

Numerical and Source Data

Proteomic analyses

Supplementary File 1 contains the peptide sequences data of the mitochondrial proteomics (tab 1). Supplementary File 1 also summarizes the log_2 protein ratios, *p* values, and sequences counts for every protein identified in the control and experimental conditions of the APEX and BirA* experiments (tabs 2 and 3). These data were used to generate the volcano plots in Figure 2A-B. Additionally, the proteome data have been uploaded onto the http://toxodb.org website, and includes an interactive search tool to enable users to search the *T. gondii* genome for genes encoding mitochondrial proteins (see Lines 1008-1018 for a description).

Supplementary File 5 lists the fold change and peptide intensities for each protein identified in the proteomic analyses of the *Tg*ApiCox25 and *Tg*Tom40 immunoprecipitations. These data were used to generate the volcano plot in Figure 6C.

Mitochondrial proteome analyses

Supplementary File 1 contains a list of the phenotype scores, MitoProt predictions, orthology grouping and a summary of the BLAST analyses (tabs 5 and 6) used to calculate the data from Figure 2, Figure 2 – figure supplement 1, and Figure 4. Supplementary File 3 provides a summary of the individual proteins identified in the construction of Figure 2D.

Model definitions

Lines 997-1001. Description of the linear mixed-effects models used in analyzing the XFe96 data, including definitions of the random and fixed effects. The R script used for this analysis is presented in Source Code File 1.

Codes used in data analysis

We include the R scripts used in analyzing the XFe96 data (Source Code File 1), and the proteomic data from the *Tg*ApiCox25/*Tg*Tom40 immunoprecipitations (Source Code File 2).

eLife Sciences Publications, Ltd is a limited liability non-profit non-stock corporation incorporated in the State of Delaware, USA, with company number 5030732, and is registered in the UK with company number FC030576 and branch number BR015634 at the address 1st Floor, 24 Hills Road, Cambridge CB2 1JP | August 2014
